# The major factor of left ventricular systolic dysfunction in patients with cardiac amyloidosis: Amyloid overload or microcirculation impairment?

**DOI:** 10.3389/fcvm.2023.1096130

**Published:** 2023-01-26

**Authors:** Jianyao Lu, Peijun Zhao, Jinhan Qiao, Zhaoxia Yang, Dazhong Tang, Xiaoyue Zhou, Lu Huang, Liming Xia

**Affiliations:** ^1^Department of Radiology, Tongji Hospital, Tongji Medical College, Huazhong University of Science and Technology, Wuhan, China; ^2^The Department of MRI, First Affiliated Hospital of Xiamen University, Xiamen, China; ^3^MR Collaboration, Siemens Healthineers Ltd., Shanghai, China

**Keywords:** cardiac amyloidosis, magnetic resonance imaging, left ventricular systolic dysfunction, amyloid overload, microcirculation impairment

## Abstract

**Purpose:**

Amyloid overload and microcirculation impairment are both detrimental to left ventricular (LV) systolic function, while it is not clear which factor dominates LV functional remodeling in patients with cardiac amyloidosis (CA). The purpose of this study was to investigate the major factor of LV systolic dysfunction using cardiac magnetic resonance imaging.

**Materials and methods:**

Forty CA patients and 20 healthy controls were included in this study. The CA group was divided into two subgroups by the left ventricular ejection fraction (LVEF): patients with reduced LVEF (LVEF < 50%, rLVEF), and patients with preserved LVEF (LVEF ≥ 50%, pLVEF). The scanning sequences included cine, native and post-contrast T1 mapping, rest first-pass perfusion and late gadolinium enhancement. Perfusion and mapping parameters were compared among the three groups. Correlation analysis was performed to evaluate the relationship between LVEF and mapping parameters, as well as the relationship between LVEF and perfusion parameters.

**Results:**

Remarkably higher native T1 value was observed in the rLVEF patients than the pLVEF patients (1442.2 ± 85.8 ms vs. 1407.0 ± 93.9 ms, adjusted *p* = 0.001). The pLVEF patients showed significantly lower slope dividing baseline signal intensity (slope%BL; rLVEF vs. pLVEF, 55.1 ± 31.0 vs. 46.2 ± 22.3, adjusted *p* = 0.001) and a lower maximal signal intensity subtracting baseline signal intensity (MaxSI-BL; rLVEF vs. pLVEF, 43.5 ± 23.9 vs. 37.0 ± 18.6, adjusted *p* = 0.003) compared to the rLVEF patients. CA patients required more time to reach the maximal signal intensity than the controls did (all adjusted *p* < 0.01). There was no significant correlation between LVEF and first-pass perfusion parameters, while significant negative correlation was observed between LVEF and native T1 (*r* = −0.434, *p* = 0.005) in CA patients.

**Conclusion:**

Amyloid overload in the myocardial interstitium may be the major factor of LV systolic dysfunction in CA patients, other than microcirculation impairment.

## Introduction

Systemic amyloidosis is a disease caused by insoluble, toxic amyloid precursor protein deposited in human tissues (1). Up to 50% of systemic amyloidosis patients suffer cardiac involvement in the course of the disease (2). The most common cardiac amyloidosis (CA) types are immunoglobulin light-chain amyloidosis (AL) and transthyretin amyloidosis (ATTR) (3, 4). Both AL and ATTR can result in progressive infiltration of amyloid fibrin in the heart, including extracellular matrix of the myocardium and coronary artery system, which damages the structure of the myocardium and vascular wall, then cardiac function (5).

Echocardiography is often used to monitor the cardiac morphology and function, but its application is limited due to its insufficient ability to evaluate the myocardial tissue. Cardiac magnetic resonance (CMR) imaging is not only the reference standard for noninvasive assessment of cardiac morphology and function (6), but also advantageous in quantitative myocardial tissue characterization by parametric mapping techniques, which compensates for the deficiency of traditional echocardiography. Extracellular volume (ECV) fraction, calculated by native T1 and post-contrast T1 values, represents the volume proportion occupied by non-cardiomyocyte components (7). As a result of the deposition of interstitial amyloid fibrin and its cytotoxic effect, the increased native T1 and ECV have been reported (8, 9), which reflect the degree of amyloid overload (10, 11). Furthermore, the rest first-pass perfusion sequence of CMR can semi-quantitatively evaluate myocardial perfusion, reflected by parameters as slope, maximal signal intensity (MaxSI), and time to maximal signal intensity (TTM). In addition, late gadolinium enhancement (LGE) images in CA patients are frequently positive, and the distinctive pattern is subendocardial or transmural dust-like enhancement (12).

Heart failure with preserved ejection fraction (HFpEF) is a common manifestation of CA patients, and ultimately progress to heart failure with reduced ejection fraction (HFrEF). Chest pain symptom may present in a proportion of CA patients without significant coronary arteries stenosis (<50%) by angiography, and the microcirculation impairment of CA patients was previously reported (13–16). In severe cases, it can lead to filling defect on first-pass perfusion images (17, 18). Amyloid overload and microcirculation impairment can both negatively affect left ventricular (LV) function, they represent two distinct mechanisms and pathways. Currently, it is not clear which factor dominates LV functional remodeling in CA patients, and few previous studies focused on patients’ myocardial perfusion. Accordingly, the purpose of our study is to evaluate amyloid overload and microcirculation perfusion of CA patients, and to further investigate the question of which factor, amyloid overload or microcirculation impairment, dominates the LV systolic dysfunction.

## Materials and methods

### Study population

Our study was approved by the Institutional Review Board of our hospital, and informed consent was waived due to the retrospective design.

Patients confirmed CA who underwent CMR examinations in our department from January 2016 to March 2021 were retrospectively enrolled. Inclusion criteria were as follows (19): (1) a biopsy of the heart or extracardiac tissue (kidney, fat, and bone marrow) with a demonstration of positive Congo red staining and apple green appeared under polarization, and (2) typical cardiac amyloidosis features on CMR images. Exclusion criteria included (1) typical myocardial infarction images on LGE, or coronary angiography indicated significant epicardial coronary artery stenosis (≥ 50%), or (2) history of myocarditis or other cardiomyopathies, or (3) poor image quality of CMR.

According to LVEF, CA patients were divided into two subgroups: CA with reduced LVEF (rLVEF, LVEF < 50%), and CA with preserved LVEF (pLVEF, LVEF ≥ 50%). We also included 20 healthy controls with matched sex and age, who were selected from our database of healthy volunteers (20). None of them had history or symptom of cardiovascular diseases, with normal electrocardiography, echocardiography and CMR.

Patients’ clinical history, the symptom-CMR interval (the time interval from the onset of heart failure symptoms to the first CMR examination) and serological examinations, were collected from digital medical record system.

### Cardiac magnetic resonance examination

All patients underwent CMR examinations on a 3T MR scanner (MAGNETOM Skyra, Siemens Healthcare, Erlangen, Germany). CMR scanning protocols included cine, T1 mapping (native and post-contrast), rest first-pass perfusion and LGE. Cine images were acquired in 4-chamber and short-axis slices using a steady-state free precession sequence on the end-expiratory breath-hold (field of view [FOV]: 360 mm × 360 mm; repetition time [TR]/time to echo [TE]/flip angle: 2.5 ms/1.4 ms/55°; slice thickness: 8 mm; voxel size: 1.9 mm × 1.9 mm × 8.0 mm; bandwidth: 965 Hz/pixel). Native and post-contrast T1 mapping images were acquired using a single breath-hold MOLLI sequence with 5b(3b)3b (b for heartbeat) and 4b(1b)3b(1b)2b acquisition schema, respectively (TR/TE/flip angle: 3.8 ms/1.2 ms/35°; voxel size: 1.4 mm × 1.4 mm × 5.0 mm, slice thickness: 5 mm). Rest first-pass perfusion images were acquired with intravenous injection of gadobenate dimeglumine (MultiHance; 0.5 mmol/ml; Bracco, Milan, Italy) at a dose of 0.2 mmol/kg and a flow rate of 2.5 ml/s (TR/TE/flip angle: 2.03 ms/1.03 ms/10°; slice thickness: 8 mm). Three short-axis images (basal, middle, and apical) and one 4-chamber image were completed in 60 cardiac cycles.

### Image post-processing

All CMR images were transferred to an offline commercial cardiac analysis software (CVI42, Circle Cardiovascular Imaging, Calgary, Canada). Two radiologists (JL and JQ with 3 and 4 years of CMR experience respectively), performed all CMR images post-processing blinded to all identifying information of patients.

The endo- and epicardial contours at the end-diastolic and end-systolic phases on CMR cine images were automatically detected and manually corrected to compute the cardiac functional and morphologic parameters, including LVEF, cardiac output index (CI) and left ventricular end-diastolic and end-systolic volume index (LVEDVi, LVESVi), LV mass index (LVMi), as well as left ventricular end-diastolic maximal wall thickness (LVMWT).

Global native T1 value, post-contrast T1 value and ECV were measured. The ECV calculation formula was as follow:


ECV=(1−HCT)×1T1postcontrastmyo−1T1nativemyo1T1postcontrastblood−1T1nativeblood

**Figure 1:**
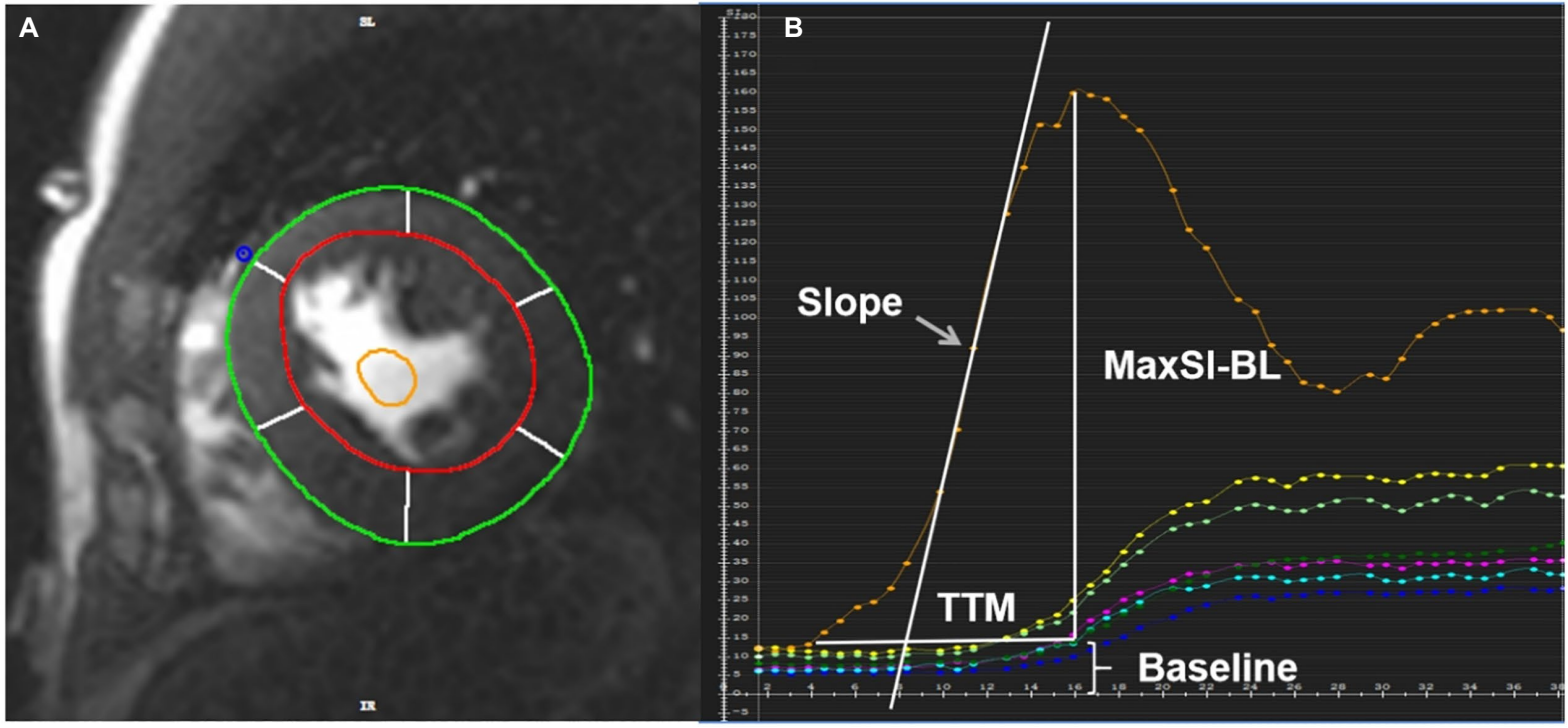
**(A)** The region of interest of the rest first-pass images was plotted in the middle myocardium. The red circle represented the LV endocardial contour with trabeculation and papillary muscles excluded, the green circle represented the LV epicardial contour, the yellow circle represented the blood pool contour. **(B)** Slope, TTM, MaxSI and baseline signal intensity can be obtained from the time-signal intensity curve of myocardium and blood pool. The orange curve represents blood pool signal, and the others represent myocardial signal. TTM, time to maximum signal intensity; MaxSI, maximal signal intensity; MaxSI-BL, maximal signal intensity subtracting baseline signal intensity.

In the evaluation of rest first-pass perfusion, the endo- and epicardial as well as blood pool contours at the basal, middle, and apical segments were drawn (Figure 1A). Slope, TTM, MaxSI and baseline signal intensity (BL) were derived from the myocardium and blood pool time-signal intensity curve (Figure 1B). We used slope dividing BL (slope%BL), MaxSI subtracting BL (MaxSI-BL) and TTM as semi-quantitative parameters of myocardial perfusion.

**Figure 2:**
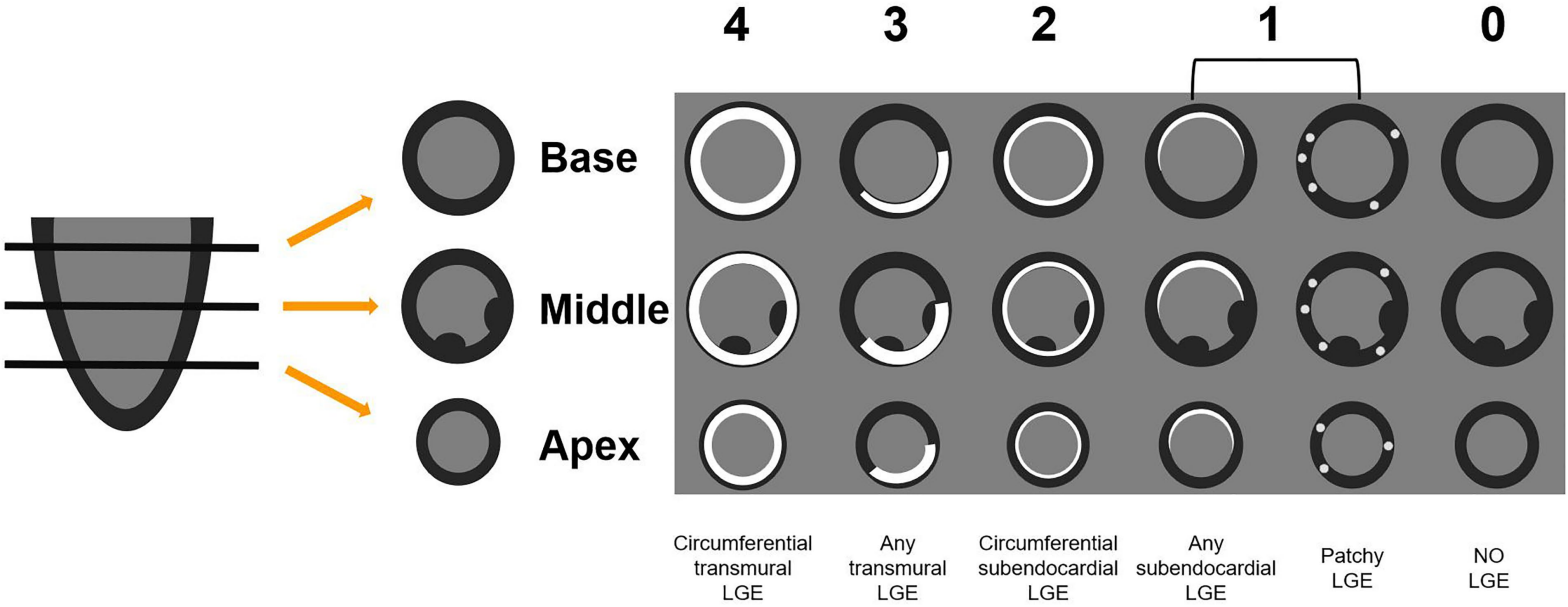
The QALE score system detailed grading rule. Circumferential transmural LGE is scored as 4; if there is any transmural LGE, then the score is 3. Circumferential subendocardial LGE is scored as 2; any subendocardial or patchy LGE is scored as 1. No LGE is scored as 0. The global left ventricular LGE score was calculated by adding the scores of the basal, mid-ventricular and apical segments. QALE, query amyloid late enhancement; LGE, late gadolinium enhancement.

Due to the lack of contrast to normal myocardium, LGE images of CA patients generally had poor image quality, and quantitative evaluation of LGE was error-prone. We therefore adopted a semi-quantitative assessment method, Query Amyloid Late Enhancement (QALE) score system, proposed by Dungu et al. (21). Scoring details were provided in Figure 2. LGE images were scored by two observers (JL and JQ) independently. A senior radiologist (LH, with 10 years of CMR experience) was assigned to adjudicate if there was any discrepancy between the scores of the two observers.

### Statistical analysis

Statistical analysis was performed by using IBM SPSS statistics version 26.0 (Chicago, IL, United States). Categorical data were reported in frequency (percentages), which were compared between groups using the Chi-square test, or the Fisher test when the expected cell count was less than five. Continuous data were presented as mean ± standard deviation (SD) or median [interquartile range]. Continuous data among the three groups were compared using one-way analysis of variance (ANOVA) or the Kruskal–Wallis test as appropriate. The Bonferroni correction was applied for post-hoc comparisons. For the comparison of some baseline clinical features of CA patients, independent *t*-tests and Mann–Whitney *U*-tests were applied. Correlation analysis was carried out using the Pearson or Spearman correlation. *p* < 0.05 was considered statistically significant.

## Results

### Baseline characteristics

Forty confirmed CA patients (age 59 ± 9 years old, and 65% male) were finally enrolled in this study, including 38 cases of AL and two cases of ATTR. The CA group were divided into two subgroups, including rLVEF (*n* = 18) subgroup and pLVEF (*n* = 22) subgroup. Twenty healthy controls (age 58 ± 8 years old, and 65% male) were also enrolled. Table 1 showed the baseline characteristics of all subjects. No significant difference was observed in the level of peak high sensitivity cardiac troponin I (hs-cTnI), the pericardial effusion rate, the pleural effusion rate and cardiac symptom rate between rLVEF patients and pLVEF patients. The level of peak N-terminal prohormone of brain natriuretic peptide (NT-proBNP) of rLVEF patients was markedly elevated compared to pLVEF patients (5703.5 [2682.5, 8136.5] pg/ml vs. 1659.5 [492.5, 4574.5] pg/ml, *p* = 0.024).

**Table 1:** Baseline characteristics of 40 CA patients and 20 healthy controls.

Characteristic	Control (*n* = 20)	CA with pLVEF (*n* = 22)	CA with rLVEF (*n* = 18)	*p*-Value
Age, year	58 ± 8	62 ± 8	57 ± 10^§^	0.111
Male, *n* (%)	13 (62)	16 (73)	10 (56)	0.516
Height, m	1.65 ± 0.07	1.67 ± 0.08	1.64 ± 0.08	0.540
Weight, kg	63.9 ± 9.7	62.1 ± 9.4	60.8 ± 12.4	0.645
BSA, m^2^	1.70 ± 0.14	1.69 ± 0.16	1.66 ± 0.18	0.680
Heart rate, beats/min	70 ± 15	79 ± 13^*^	86 ± 13^*^	0.002
Symptom-CMR interval, month	–	5 [1, 12]	6 [1.5, 11.8]	–
Systolic BP, mmHg	119 ± 25	116 ± 20	114 ± 22	0.791
Diastolic BP, mmHg	77 ± 13	74 ± 14	75 ± 13	0.718
Peak level hs-cTnI, pg/ml	–	71.1 [17.4, 218.1]	53.6 [23.3, 174.7]	–
Peak NT-proBNP, pg/ml	–	1659.5 [492.5, 4574.5]	5703.5 [2682.5, 8136.5]^§^	–
Hematocrit, %	39.1 ± 4.1	36.0 ± 5.5	34.4 ± 7.1^*^	0.037
Pericardial effusion, *n* (%)	–	18 (82)	17 (94)	–
Pleural effusion, *n* (%)	–	18 (82)	13 (72)	–
Palpitation, *n* (%)	–	3 (14)	4 (22)	–
Chest distress, *n* (%)	–	12 (55)	13 (72)	–
Chest pain, *n* (%)	–	1 (5)	2 (11)	–
E/A ratio ≥ 2.0, *n* (%)	–	5 (23)	8 (44)	–

* Compared to the control group, adjusted *p* < 0.05.

§ Compared to the CA with pLVEF group, adjusted *p* < 0.05. *p*-Value referred to the overall comparison among three groups.

CA, cardiac amyloidosis; rLVEF, reduced left ventricular ejection fraction; pLVEF, preserved left ventricular ejection fraction; BSA, body surface area; CMR: cardiac magnetic resonance; BP, blood pressure; NT-proBNP, N-terminal pro-brain natriuretic peptide.

### Left ventricular morphologic and functional features

With respect to LV morphology, the LVEDVi and LVESVi of rLVEF patients were markedly higher than pLVEF patients and controls, however, there was no significant difference between pLVEF patients and controls in LV volume. The LVMWT and LVMi of CA patients were notably higher than that of controls (Table 2).

**Table 2:** Morphologic and functional CMR data of all study groups.

Characteristic	Control (*n* = 20)	CA with pLVEF (*n* = 22)	CA with rLVEF (*n* = 18)	*p*-Value
LVEDVi, ml/m^2^	73.1 ± 12.5	71.2 ± 17.5	86.5 ± 23.4^*^^,^^§^	0.022
LVESVi, ml/m^2^	23.7 ± 8.5	31.7 ± 18.8	56.5 ± 20.0^*^^,^^§^	<0.001
LVMWT, mm	9.7 ± 1.3	16.4 ± 3.1^*^	14.8 ± 2.13^*^	<0.001
LVMi, g/m^2^	43.3 ± 7.8	75.5 ± 21.8^*^	81.9 ± 22.9^*^	<0.001
LVEF, %	67.3 ± 10.2	59.4 ± 6.2^*^	35.1 ± 9.1^*^^,^^§^	<0.001
CI, L•min^−1^/m^2^	3.4 ± 1.0	3.3 ± 0.7	2.5 ± 0.8^*^^,^^§^	0.007

* Compared to the control group, adjusted *p* < 0.05.

§ Compared to the CA with pLVEF group, adjusted *p* < 0.05. *p*-Value referred to the overall comparison among three groups.

CA, cardiac amyloidosis; rLVEF, reduced left ventricular ejection fraction; pLVEF, preserved left ventricular ejection fraction; CMR, cardiac magnetic resonance; LVEDVi, left ventricular end-diastolic volume index; LVESVi, left ventricular end-systolic volume index; LVMWT, left ventricular maximal wall thickness; LVMi, left ventricular mass index; CI, cardiac index.

A deceasing trend from controls to pLVEF patients and then to rLVEF patients in respect of LVEF could be observed (all adjusted *p* < 0.05). The CI of rLVEF patients was significantly decreased than that of pLVEF patients and controls.

### Left ventricular tissue and perfusion features

As illustrated in Table 3, the LV native T1 and ECV of two CA groups were all markedly higher than that of the controls (all adjusted *p* < 0.001). Higher native T1 value was observed in rLVEF group than pLVEF group (1442.28 ± 5.8 ms vs. 1407.0 ± 93.9 ms, adjusted *p* = 0.001), but there was no significant difference with regard to ECV and LGE scores between the two case groups.

**Table 3:** Rest first-pass perfusion and tissue characteristics.

Characteristic	Control (*n* = 20)	CA with pLVEF (*n* = 22)	CA with rLVEF (*n* = 18)	*p*-Value
Native T1, ms	1223.3 ± 62.6	1407.0 ± 93.9^*^	1442.2 ± 85.8^*^^,^^§^	<0.001
ECV, %	25.7 ± 3.5	47.7 ± 11.5^*^	46.6 ± 9.1^*^	<0.001
LGE score	–	5.7 ± 3.6	5.4 ± 3.6	–
Slope%BL	52.9 ± 17.9	46.2 ± 22.3^*^	55.1 ± 31.0^§^	<0.001
TTM, s	32.3 ± 14.9	44.9 ± 17.9^*^	46.2 ± 15.3^*^	<0.001
MaxSI-BL	40.1 ± 17.2	37.0 ± 18.6	43.5 ± 23.9^§^	0.003

* Compared to the control group, adjusted *p* < 0.05.

§ Compared to the CA with pLVEF group, adjusted *p* < 0.05. *p*-Value referred to the overall comparison among three groups.

rLVEF, reduced left ventricular ejection fraction; pLVEF, preserved left ventricular ejection fraction; ECV, extracellular volume; LGE, late gadolinium enhancement; Slope%BL, slope dividing baseline signal intensity; TTM, time to maximal signal intensity; MaxSI-BL, maximal signal intensity subtracting baseline signal intensity.

The pLVEF patients exhibited a lower global slope%BL (46.2 ± 22.3 vs. 55.1 ± 31.0, adjusted p = 0.001) and lower MaxSI-BL (37.0 ± 18.6 vs. 43.5 ± 23.9, adjusted *p* = 0.003) than rLVEF patients. There was no significant difference in TTM between rLVEF patients and pLVEF patients. The TTM of CA patients was much longer than that of the controls (adjusted *p* < 0.001). No significant difference was found between rLVEF patients and controls with respect to Slope%BL and MaxSI-BL.

**Figure 3:**
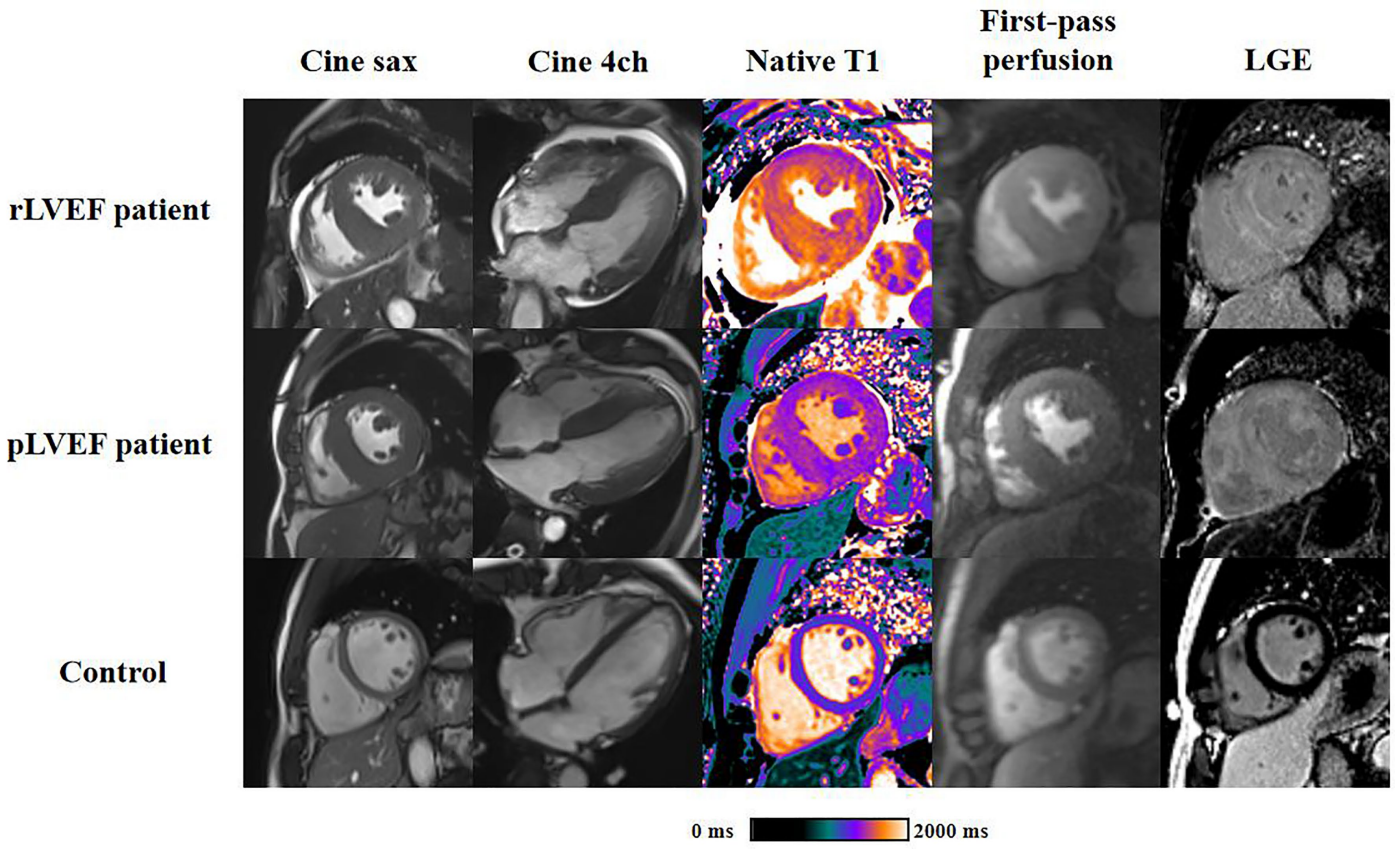
Representative cardiac magnetic resonance plots of rLVEF patient, pLVEF patient and control. Images from left to right are end-diastolic short-axis cine, end-diastolic 4-chamber cine, native T1 mapping, end-diastolic first-pass perfusion and late gadolinium enhancement, respectively. All short-axis images are acquired in middle segment. rLVEF, reduced left ventricular ejection fraction; pLVEF, preserved left ventricular ejection fraction; sax, short-axis; 4ch, 4-chamber; LGE, late gadolinium enhancement.

Representative CMR images of three groups were shown in Figure 3.

### Correlation analysis

**Figure 4:**
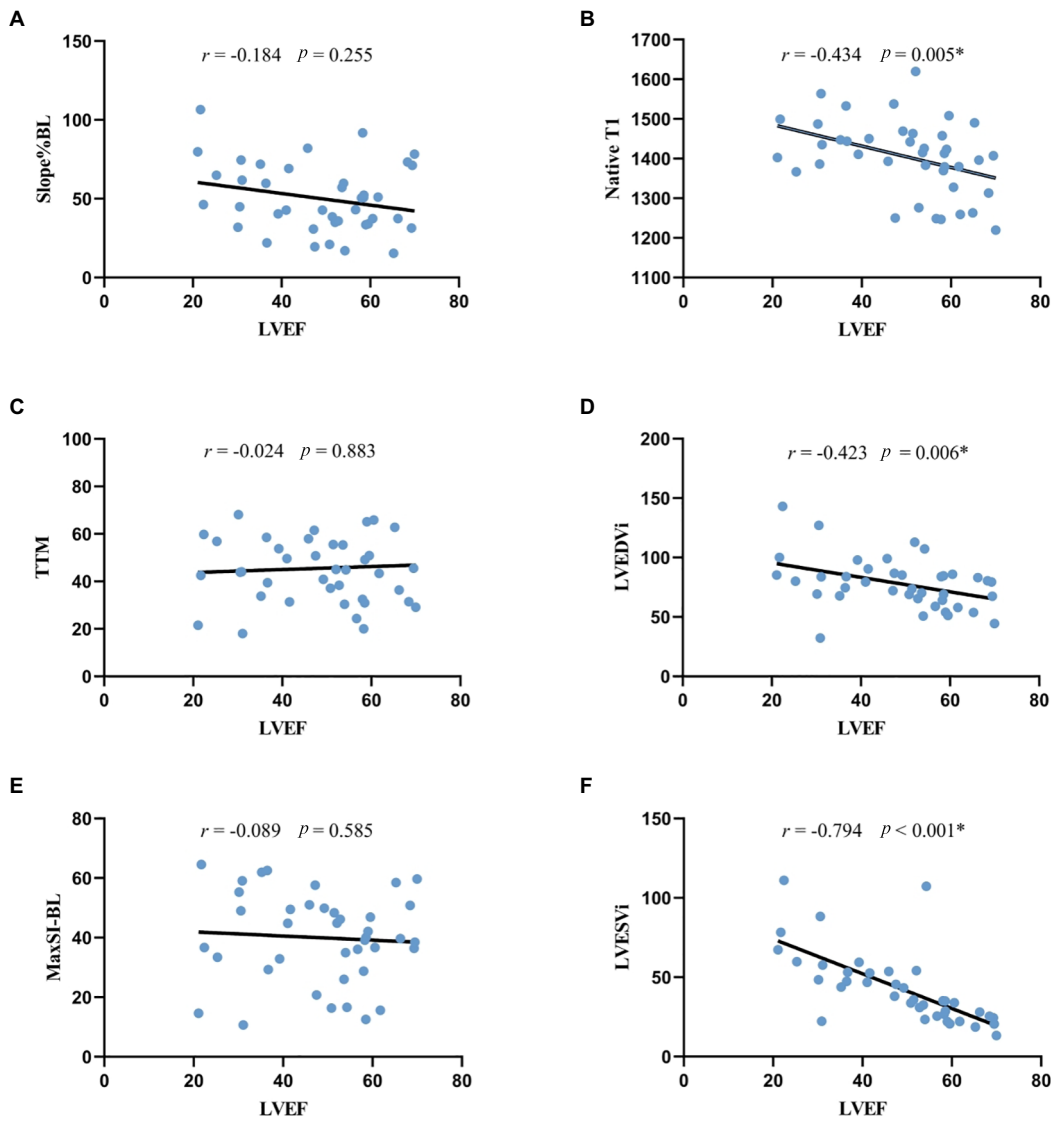
Correlation analysis between LVEF and morphologic, perfusion and quantitative mapping parameters. *p < 0.05. **(A,C,E)** the correlation between LVEF and rest first-pass perfusion parameters, indicating the negative correlation was not statistically significant. **(B)** the negative correlation between LVEF and native T1 was statistically significant. **(D,F)** LVEF was significantly correlated with LV morphologic parameters. LVEF, left ventricular ejection fraction; Slope%BL, slope dividing baseline signal intensity; TTM, time to maximal signal intensity; MaxSI-BL, maximal signal intensity subtracting baseline signal intensity; LVEDVi, left ventricular end-diastolic volume index; LVESVi, left ventricular end-systolic volume index.

As shown in scatter plots (Figure 4), the native T1, LVEDVi and LVESVi presented statistically significant negative correlations with LVEF (all *p* < 0.05). Global slope%BL, MaxSI-BL, and TTM showed insignificant correlation with LVEF (slope%BL: *r* = −0.184, *p* = 0.255; MaxSI-BL: *r* = −0.089, *p* = 0.585; TTM: *r* = −0.024, *p* = 0.883).

## Discussion

In this study, we used CMR to assess amyloid burden and myocardial microcirculation in 40 CA patients. The major findings were: (1) the amyloid burden of rLVEF patients was heavier than that of pLVEF patients, and native T1 significantly correlated with LVEF, which suggests that there is a tight link between the increasing of amyloid burden and the worsening of LV dysfunction; (2) no significant correlation was observed between LVEF and first-pass perfusion parameters, indicating that the microcirculation impairment has a minor role in LV dysfunction; (3) the LV functional remodeling and the LV morphologic remodeling of CA patients were closely related.

The most obvious finding to emerge from the present study is that amyloid overload is a major factor of LV systolic dysfunction in CA patients. Amyloid-mediated LV systolic dysfunction is secondary to impaired calcium handling (22). It can also produce cardiotoxicity by interfering with the mitochondrial membrane potential and impairing the lysosomal function (23, 24). The negative effect on LV systolic function is enhanced by the superposition of direct injury and cardiotoxicity, and the cardiac functional manifestation could change from HFpEF to HFrEF.

The microcirculation impairment in CA patients was found in this study, and we speculated that it is mainly caused by intramural small vessel lesions instead of epicardial coronary artery lesion. Amyloid fibrin deposited in endothelial cells and smooth muscle cells of small arteries leads to proliferation of the medial components and then to inward hypertrophic (25, 26). And the external compression of the vessels by extracellular amyloid deposition also drives perfusion impairment (27). The epicardial coronary artery is larger in diameter than small intramural blood vessels, so it is influenced by amyloid deposition to a lesser extension.

There are two unexpected findings: First, the Slope%BL and MaxSI-BL of rLVEF patients showed no significant difference compared to the controls; Second, the pLVEF patients showed worse microcirculation perfusion than the rLVEF patients. Some previous studies may help explain them. Sharma et al. (28) reported that the intramural vessel lumen area of CA patients was larger than that of control (CA patients vs. Control, 1,518 μm^2^ vs. 439 μm^2^), it was considered as a compensating change (positive remodeling) caused by amyloid deposition, which could be a reasonable interpretation of the first above-mentioned result. The positive remodeling is common with acute coronary syndrome patients, it relates to large plaque area and complex vessel lesions and is considered as a mechanism for delaying the disease progression (29, 30). As to the second result, the insignificant correlation between LVEF and perfusion parameters proposed in our study can explain it to some extent, and this result also reflects the possibility that the deposition of amyloid fibrin is locally uneven. Modesto et al. (26) proposed that the intima-media thickening and dysfunction of peripheral arteries could be observed in primary amyloidosis patients, but the cardiac involvement was not necessarily accompanied. Mueller et al. performed autopsy examinations on 11 CA patients with severe intramural vessel obstruction but no significant epicardial coronary arteries stenosis. They found amyloid deposition in myocardial interstitium was absent in 3 of the subjects (31). Hosch et al. (25) performed anatomical dissection on 5 CA patients’ heart, found the severity of myocardial interstitial involvement was inconsistent with the severity of intramural vessels involvement. And CA patients with intramural vessels involvement only were also reported before (32).

A few previous studies focused on similar extent of the present research. Dorbala et al. (15) performed quantitative analysis of myocardial perfusion on 21 CA patients without coronary artery disease using positron emission tomography (PET), their results revealed that CA patients had lower myocardial blood flow and coronary flow reserve than hypertensive LV hypertrophy patients. And the correlation between microcirculation perfusion and LVEF was insignificant, which is in accordance with the present results. Li et al. (16) expounded that there was a positive relationship between first-pass perfusion parameters and the myocardial wall thickening of AL patients, which is inconsistent with our results and the reasons could be as follow: the study population were both limited (Li: *n* = 32, this study: *n* = 40); and the study design were both retrospective, it is possible that there was a patient selection bias; what is more, the amyloid burden of Li’s subjects was under-explored as they did not include CMR mapping parameters and LGE information, which neglected the effect of amyloid burden on cardiac function.

Our results showed that the native T1 of rLVEF patients is higher than that of pLVEF patients, but there was no difference in ECV between the two groups. The possible explanation for this is as follows: Native T1 reflects not only extracellular changes, but also intracellular changes. Myocardial edema, caused by the cardiotoxicity of amyloid fibrin, can lead to the elevating of native T1 value. However, the increasing in ECV caused by intracellular edema is almost negligible. Native T1 reflects two parts of pathological changes in CA patients, while ECV focuses on just one of them. That might be the reason why the native T1 showed difference between rLVEF and pLVEF patients while ECV did not. In addition, the numerical value of native T1 is relatively larger than ECV, which may contribute native T1 a more sensitive parameter to reflect the amyloid load of CA patients.

The significant negative correlation between LVEF and LVEDVi/LVESVi showed the tight link between LV functional remodeling and LV morphologic remodeling. The volume load gradually increases as the cardiac function decreases, inducing serial addition of sarcomeres to accommodate the greater ventricular volumes and subsequent ventricular dilatation (33).

Our study has several limitations. First, due to the low incidence of CA and the restriction of inclusion criteria, the sample of our study is relatively small, and more patients need to be included in the future to further verify the current findings. Furthermore, because of the skewed proportion of ATTR and AL in this study, we cannot subdivide them and compare the two types of CA patients.

## Conclusion

The overload of amyloid fibrin in myocardial interstitium is significantly associated with adverse LV functional remodeling in CA patients, while microcirculation impairment shows no significant correlation with LVEF, suggesting that it may play a minor role in LV systolic dysfunction.

## Data availability statement

The original contributions presented in the study are included in the article/supplementary material, further inquiries can be directed to the corresponding author.

## Ethics statement

The studies involving human participants were reviewed and approved by the Ethical Committee of the Tongji Medical College, Huazhong University of Science and Technology. Written informed consent for participation was not required for this study in accordance with the national legislation and the institutional requirements.

## Author contributions

Study design was performed by JL. Image analysis and statistical computation were performed by JL and JQ. The first draft of the manuscript was written by JL. Suggestions for revision were proposed by LH, LX, PZ, ZY, and XZ. MR scanning was carried out by DT. All authors contributed to the article and approved the submitted version.

## Funding

This study was supported by a grant from the National Natural Science Foundation of China (No. 81873889).

## Conflict of interest

XZ is employed by Siemens Healthineers Ltd.

The remaining authors declare that the research was conducted in the absence of any commercial or financial relationships that could be construed as a potential conflict of interest.

## Publisher’s note

All claims expressed in this article are solely those of the authors and do not necessarily represent those of their affiliated organizations, or those of the publisher, the editors and the reviewers. Any product that may be evaluated in this article, or claim that may be made by its manufacturer, is not guaranteed or endorsed by the publisher.
